# A Multiscale Investigation on the Thermal Transport in Polydimethylsiloxane Nanocomposites: Graphene vs. Borophene

**DOI:** 10.3390/nano11051252

**Published:** 2021-05-11

**Authors:** Alessandro Di Pierro, Bohayra Mortazavi, Hamidreza Noori, Timon Rabczuk, Alberto Fina

**Affiliations:** 1 Dipartimento di Scienza Applicata e Tecnologia, Politecnico di Torino, Alessandria Campus, Viale Teresa Michel 5, 15121 Alessandria, Italy; dipierro.alessandro@gmail.com; 2 Department of Mathematics and Physics, Leibniz Universität Hannover, Appelstraße 11, 30167 Hannover, Germany; 3 Institute of Structural Mechanics, Bauhaus-Universität Weimar, Marienstr. 15, 99423 Weimar, Germany; hamidr_noori@yahoo.com (H.N.); timon.rabczuk@uni-weimar.de (T.R.)

**Keywords:** borophene, graphene, polydimethylsiloxane, interfacial thermal conductance, thermal conductivity, nanocomposites, multiscale modelling

## Abstract

Graphene and borophene are highly attractive two-dimensional materials with outstanding physical properties. In this study we employed combined atomistic continuum multi-scale modeling to explore the effective thermal conductivity of polymer nanocomposites made of polydimethylsiloxane (PDMS) polymer as the matrix and graphene and borophene as nanofillers. PDMS is a versatile polymer due to its chemical inertia, flexibility and a wide range of properties that can be tuned during synthesis. We first conducted classical Molecular Dynamics (MD) simulations to calculate the thermal conductance at the interfaces between graphene and PDMS and between borophene and PDMS. Acquired results confirm that the interfacial thermal conductance between nanosheets and polymer increases from the single-layer to multilayered nanosheets and finally converges, in the case of graphene, to about 30 MWm^−2^ K^−1^ and, for borophene, up to 33 MWm^−2^ K^−1^. The data provided by the atomistic simulations were then used in the Finite Element Method (FEM) simulations to evaluate the effective thermal conductivity of polymer nanocomposites at the continuum level. We explored the effects of nanofiller type, volume content, geometry aspect ratio and thickness on the nanocomposite effective thermal conductivity. As a very interesting finding, we found that borophene nanosheets, despite having almost two orders of magnitude lower thermal conductivity than graphene, can yield very close enhancement in the effective thermal conductivity in comparison with graphene, particularly for low volume content and small aspect ratios and thicknesses. We conclude that, for the polymer-based nanocomposites, significant improvement in the thermal conductivity can be reached by improving the bonding between the fillers and polymer, or in other words, by enhancing the thermal conductance at the interface. By taking into account the high electrical conductivity of borophene, our results suggest borophene nanosheets as promising nanofillers to simultaneously enhance the polymers’ thermal and electrical conductivity.

## 1. Introduction

Thermal management is a design issue in several fields, such as electronics and electrified vehicles. In fact, inappropriate thermal management may result in the sudden or local rise of hot spots, which not only can substantially shorten a device’s life but can also lead to hazardous operating conditions. In recent years, the development of flexible devices, such as wearable technologies, have presented new challenges in thermal management, as traditional solutions are often less effective. After graphene was successfully isolated in 2004 [1,2], two-dimensional (2D) materials started to attract tremendous attention. Graphene exhibits outstanding physical properties, including ultra-high thermal, mechanical and electronic carrier mobility. The high-quality properties of graphene, along with its outstanding flexibility, have made this nanomaterial a highly promising candidate for employment in thermal management systems [3]. Graphene continues to attract large interest among researchers [4] due to its electronic structure, which reflects outstanding thermal properties and has a calculated thermal conductivity up to 10 KW m^−1^ K^−1^ [5]. Within 2D materials, buckled borophene [6,7] monolayer was synthetized in 2015 by Mannix et al. [8] and was immediately perceived as a graphene competitor. Despite a thermal conductivity lower than graphene by a significant order of magnitude [9], borophene has attracted the interest of a large number of researchers due to the high stiffness elastic modulus and appealing electronic, thermal and conducting properties [10]. The most popular approach to exploit the exciting properties of 2D materials is to disperse them inside a continuous matrix made of polymer, creating a nanocomposite material. Although the thermal conductivity of graphene polymer nanocomposites has been extensively studied in the literature [11,12], borophene polymer nanocomposites are basically unexplored, either theoretically or experimentally. In order to improve the knowledge with respect to the application of borophene in thermal management systems, in this work we conduct a multi-scale theoretical study to compare graphene and borophene for employment as reinforcement nanomaterials for improvement of the thermal conductivity of polymeric materials. Polydimethylsiloxane (PDMS) was chosen among other polymers because it has been reported to exhibit a strong interaction with graphene [13]. Moreover, based on previous reports, PDMS was found application-relevant for its set of properties, such as a low glass transition temperature (thus flexibility), chemical inertia, and a wide range of physical states, from liquid to rubbery state, which reflect a large variety of applications. The improvement of thermal conductivity in PDMS matrix composites thus represent an alluring solution for heat transfer for several practical uses, such as highly deformable electronic devices. The thermal conductivity of PDMS and graphene composites was experimentally investigated by several authors, with findings strongly correlated to the specific structure. Zhao and colleagues [14] found that a 0.7% wt. graphene content can more than double the thermal conductivity of PDMS-based composite, from 0.19 W m^−1^ K^−1^ for pure PDMS to about 0.45 W m^−1^ K^−1^ for graphene sheet composite. Such remarkable improvement was attributed to the creation of a tight percolation network of graphene platelets; in percolation theory, above the percolation threshold the thermal conductivity boosts, determining a bi-linear trend [15,16]. The platelets employed from Zhao et al. [14] were obtained by foaming, a technique that allows the creation of particles with length in the range of some micrometers and a thickness of about 3 nm. Later, Tian et al. [17] adopted silicone rubber (SR), with three different graphene platelet concentrations obtained by mechanical blending and curing. The platelets were about 3 nm thick and about 5 by 10 micrometers in lateral size. The maximum concentration of graphene platelets, 0.72% in weight, determined an increase of the thermal conductivity from 0.2 W m^−1^ K^−1^ for neat SR to 0.3 W m^−1^ K^−1^ for the composite. The comparison between the work of Zhao [14] and Tian [17] points out how the particle displacement, and consequently interaction between particles, impacts the composite thermal conductivity. Li et al. [18] reviewed research works on graphene materials featuring oriented particles, three-dimensional structures or segregated particles. They showed that the thermal conductivity enhancement in three-dimensional structures is about five times that of segregated structures. Despite this consideration, high thermal conductivity materials made of segregated particles, such as the thermoplastics studied by Alam et al. [19] or the epoxy resin of Balandin et al. [12,20], has the drawback of filler loadings in the order of magnitude of one tenth or more, which influences significantly the compound processability.

In the present work, our objective is to explore the effectiveness of borophene as a novel and highly important nanomaterial for the enhancement of polymers’ thermal conductivity. For the sake of comparison, we compare the same structures made of graphene, representing one of the most well-performing and deeply studied materials of the last decade. For this goal we performed combined atomistic continuum multi-scale modeling. This approach follows a two-stage procedure. First, we conduced classical molecular dynamics simulations to calculate the interfacial thermal conductance (ITC: the inverse of the thermal boundary resistance [21]) that rises between the polymer and the layers of potential filler (graphene of borophene). In the next stage, the calculated interfacial thermal conductance was used as the conductance parameter between filler and matrix for the finite element method (FEM) simulations. From the FEM simulations, we were able to quantify the effective thermal conductivity of the potential polymer nanocomposites by tuning the effects of nanofiller content, thickness and aspect ratio. Our results suggest that borophene nanosheets represent a promising candidate for the improvement of polymers’ thermal conductivity.

## 2. Computational Methods

From the multi-scale modeling point of view, in order to investigate the effective thermal conductivity of nanocomposites, one needs to know the thermal conductivity of the polymer and nanosheets as well as the thermal conductance between these two phases. In this section, we first explain the conduced classical Molecular Dynamics (MD) simulations to evaluate the thermal conductance between nanosheets and PDMS. Taking the values for the thermal conductivity of pristine nanosheets from the literature, we will then discuss the continuum FEM simulations. To perform the MD simulations, the Large-scale Atomic-Molecular Massively Parallel Simulator (LAMMPS) [22,23] package was used. All the interatomic forces within the PDMS polymer were calculated by the COMPASS force field [24], a forcefield that provides a detailed representation of bond and non-bond interaction for soft matter and that is already known for PDMS in thermal applications [25]. As the most accurate choice to study the thermal conductivity of graphene, the optimized Tersoff [26] was employed to define the interactions within graphene carbon atoms. It should be noted that borophene shows various structures, and in this work, we consider buckled borophene as synthetized by Mannix et al. [8]. The interactions between boron atoms in the borophene were defined by the ReaxFF [27], which is also a reactive force field and thus is capable of managing chemical reactions. The validity of ReaxFF for the modelling of thermal transport in buckled borophene has been already been confirmed in the previous work by Mortazavi et al. [6]. The interactions between PDMS and graphene or borophene were modelled by Lennard-Jones (LJ) potential and a 10 Å cutoff was defined with all the combined pair interactions for Si, O, H, sp^2^ C, sp^3^ C and B. The equilibrium distance and potential energy well depth were calculated by applying Lorentz-Berthelot mixing rules [28] from Universal Force Field [29]. The presence of hydrogen atoms in PDMS justified a relatively small time-step of 0.25 fs. A single linear PDMS chain was made of 49 -Si-(CH_3_)_2_-O- units, Si-methyl terminated, for a total of 507 atoms. To trigger proper interactions between PDMS chains and the layered materials, PDMS molecules were packed in a larger simulation box, with PBC set in all coordinates. This system was made of 30 PDMS chains (15,210 atoms) made by a modified Markov process [30] inside a volume of about 46 × 46 × 92 Å^3^. To reach a densely packed PDMS, the system was first equilibrated using Nosé-Hoover thermostat (NVT) at 300 K and then was heated up to 500 K to allow the rearrangement of atomic position. The system was then cooled down to 300 K with the Nosé-Hoover barostat and thermostat (NPT). The final density of the polymer volume was set to 0.97 ± 0.05 g cm^−3^, to fit the typical literature values [31]. After obtaining the homogenous and bulk PDMS, the periodicity was removed along the Z direction, and multi-layer graphene or borophene films were placed on the surface of the PDMS polymer. The final models were simulated with periodic boundary conditions along all three coordinates.

Effective thermal conductivities of PDMS-based nanocomposites were obtained using FEM with ABAQUS-Standard software, along with Python subroutines for the modeling of nanocomposite samples. We evaluated the effective thermal conductivity by solving a steady-state heat transfer problem, in which load is applied by heat fluxes. The computational details are the same as those in our earlier study [32]. In these calculations, representative volume elements (RVEs)—finite parts of the volume composite—were simulated. As an acceptable assumption, the nanofillers were modelled as flat disks [33,34], dispersed randomly inside a PDMS polymer matrix. In this model, the geometry of the filler is taken into account by tuning the aspect ratio of the disks, defined as the diameter- to-thickness ratio of the disk, from 1:25 to 1:100 in four steps: 1:25, 1:50, 1:75 and 1:100. On these bases, our models cannot capture the agglomeration effect and percolation that may occur in real experimental samples; this can be an appealing topic for further studies. Within this RVE, no particle–particle contact was allowed, nor was disk bending. Heat transfer elements (DC3D4) with a 4-node linear tetrahedron shape were used in our calculations.

## 3. Results and Discussions

It should be first noted that there are several reports that state that, within layered 2D materials, the interfacial thermal conductance between nanosheets and substrate can be affected by the thickness or number of layers [35]. The aforementioned thickness dependency was predicted computationally [32] and confirmed experimentally [36,37]. A possible explanation for this finding was attributed to the progressive improvement in cross-plane phonon transmission among the low-frequency modes as the number of layers increases [35]. Therefore, the interfacial thermal conductance between PDMS and graphene or borophene nanoflakes was calculated as a function of the number of layers, from one to six. 

### 3.1. Molecular Dynamics Results

**Figure 1:**
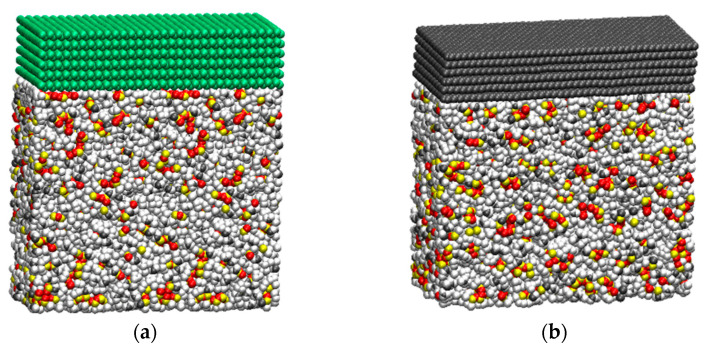
Constructed molecular models of six layers of borophene (**a**) and graphene (**b**) stacked over PDMS polymer within the simulation box cell. Color coding, includes; boron in green, carbon in dark grey, hydrogen in white, oxygen in red and silicon in yellow.

To determine the interfacial thermal conductance values for PDMS–borophene and PDMS–graphene interfaces, the asymptotic value of convergence occurring around six layers [32,35] was considered. The constructed models, with the stacked layers of graphene and borophene (six layers for both) over a block of PDMS polymer, are depicted in Figure 1. Equivalent models were used for one to five layers. These systems include periodic boundary conditions in all directions, meaning that two interfaces between nanoflakes and polymer engage in thermal transport. Nevertheless, periodic boundary conditions create a virtually continuous surface without boundaries, so the nanoflakes are designed to preserve the crystal periodicity even at the simulation box borders. The specifications for a single-layer graphene model are a = 99.64 Å; b = 47.48 Å. This is equal to a contact area of about 4731 Å^2^, which means a contact area of twice the aforementioned value form with PDMS polymer in the thermal conductance calculations. The height of the systems varied from 84.6 to 105 Å, depending on layer stacking. Each layer of graphene, 3.4 Å thick, was made of 1760 carbon atoms, bringing the total amount of atoms, including the PDMS, from 32,180 individual atoms for single layer graphene to 40,980 atoms for the six-layer model. Similarly, for the borophene models, the contact area is about 4289 Å^2^. In this case, 1260 atoms are included in each layer of borophene, bringing the total amount of atoms in the final models from 31,680 for the single layer borophene to 37,980 atoms for six-layer borophene. The height varied with borophene layer thickness; therefore, the size of composite systems along the stacking directions varied from 92 Å for the monolayer to 112 Å for the six layers of borophene. The slight difference in cell topology between graphene and borophene models is due to the lattice differences of the crystalline materials.

To calculate the thermal conductance between PDMS polymer and the layers of graphene and borophene platelets, the thermal equilibration method [32,38,39,40] was adopted. At the beginning of the simulation, atomic velocities were initialized using the Maxwell-Boltzmann distribution. During the equilibration process, the PDMS polymer and the stacked nanoplatelet temperature was kept at 300 K and 350 K, respectively, using the NVT method for 25 ps. In the second stage, we simulated the heat transfer using a transient 500 ps step, where NVT was switched off and the polymer and nanofillers were allowed to reach the thermal equilibrium in NVE, without energy exchange with the external environment. Within this latter transient stage, the temperature variation of each phase was collected. The exponential fitting of the temperature difference between the two materials allowed us to determine the decay time τ. By knowing the masses of the polymer *M_p_* and filler *M_f_*, the heat capacity of polymer *Cp_p_*, filler *Cp_f_*, and the interfacial area *A*, the interfacial thermal conductance across the interface λ was calculated by reversing Equation (1) [32,39].

(1) $$\Delta T\left(t\right)=\Delta T\left(0\right)e^{\left[-\left(\frac{1}{M_{p}Cp_{p}}+\frac{1}{M_{f}Cp_{f}}\right)\right]\lambda A}$$

**Figure 2:**
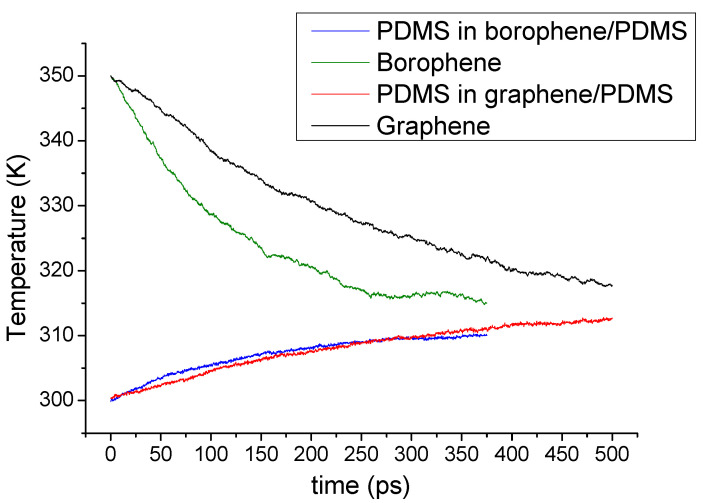
Time-averaged temperatures recorded during the heat transfer simulations for the six-layer nanosheets of graphene and borophene over the PDMS polymer.

The heat capacity values adopted in this work are literature values: 1.46 J g^−1^K^−1^ for PDMS [31], 0.71 J g^−1^K^−1^ for graphene [41] and 1.02 J g^−1^K^−1^ for borophene [7]. The interfacial thermal conductance was calculated for all systems. For graphene and borophene, the calculations were conducted for 12 uncorrelated simulations, and the temperatures were averaged. An example of thermal relaxation between borophene or graphene and PDMS is shown in Figure 2. It is noticeable that during the relaxation, the temperature difference between the nanofillers and the polymers, Δ*T(t)*, decays exponentially. By conducting a fitting to the temperature difference, the interfacial thermal conductance was evaluated using Equation (1).

**Figure 3:**
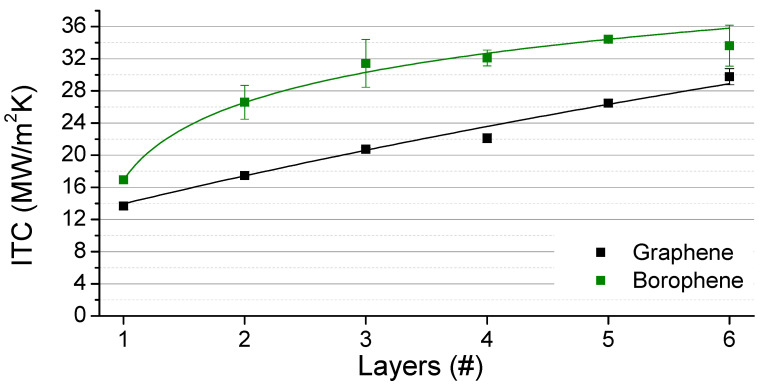
Interfacial thermal conductance (ITC) between nanosheets and PDMS as a function of the number of layers of graphene and borophene. The line guides the eye among actual values (squares).

In Figure 3, the predicted interfacial thermal conductance between graphene and borophene nanosheets and PDMS as a function of the number of layers is shown. Acquired results confirm that the interfacial thermal conductance increases from the single layer to multilayered structures. In the case of graphene, it reaches a plateau and converges to a value of around 30 MWm^−2^ K^−1^ for the six- and seven-layer graphene–PDMS systems, in agreement with the literature [32,35]. In the case of borophene, the interfacial thermal conductance sharply increases from the single-layer to three-layer structure, and for higher number of layers, it stays convincingly constant. Our results interestingly reveal that, in general, the borophene exhibits higher interfacial thermal conductance with the PDMS than graphene. In the case of borophene–PDMS, the interfacial thermal conductance converges to about 33 MWm^−2^ K^−1^, which is 10% more than that of the graphene–PDMS interface. This result shows that the buckled structure of borophene could enhance the heat transfer at the interface with the polymer even better than graphene.

### 3.2. Multi-Scale Modelling Results

**Figure 4:**
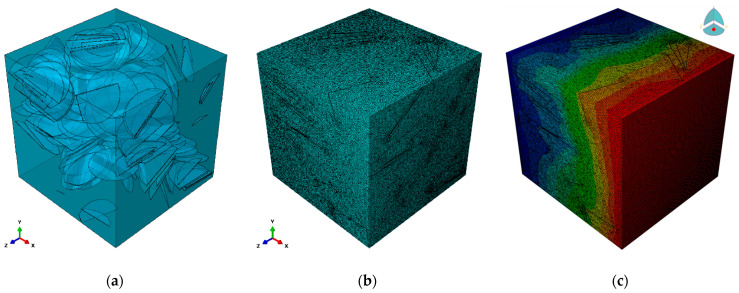
(**a**) An example of composite RVE including 150 flat disks with the aspect ratio 1:100 and 4% of volume concentration. (**b**) The four-node linear tetrahedron shape mesh for the RVE. (**c**) The established steady-state temperature profile created by applying the heat flux passing through the RVE (the color coding from red to blue depicts hot to colder regions, respectively.

The values of thermal conductance calculated by MD simulations were implemented within a continuum model to calculate the effective lattice thermal conductivity of macroscopic samples using the finite element approach. In this work, the thermal conductivity of multilayer graphene and borophene is taken from the literature and assumed to be 2000 Wm^−1^ K^−1^ [42,43] and 75 Wm^−1^ K^−1^ [6], respectively. The converged value for the interfacial thermal conductance values is used to define the thermal contact properties between the fillers and polymer. Moreover, we assumed the disc geometry for the graphene and borophene, in which the aspect ratio is defined by the diameter-to-thickness ratio. The constructed models are all periodic, meaning that if a particle crosses a boundary surface of the RVE, it enters from the opposite surface; thus, by putting the RVEs side by side, all the fillers will show the perfect disc geometry. Fillers were randomly oriented and distributed without allowing their contact, so that formation of a percolative network of conductive particles was inhibited. Three filler loadings (volume fractions) of 1%, 2% and 4% were considered perfectly dispersed within the matrix, disallowing the creation of aggregation cores, and the thickness of discs were assumed to be 1, 10 and 100 nm. In our simulations, for the evaluation of the effective thermal conductivity, a steady-state heat flux was imposed on the opposite surfaces. Within this layout, the heat flux passes through the meshed RVE and forms a temperature gradient (in the order of magnitude of 0.10 K) inside the volume, as illustrated in Figure 4. The effective thermal conductivity of PDMS-based nanocomposites was calculated, in the steady state, on the basis of one-dimensional Fourier’s law, *k_eff_* = *Lq*Δ*T*^−1^*,* where *L* is the size of RVE, *q* is the applied heat flux and Δ*T* is the established temperature difference between the two ends of the model.

**Figure 5:**
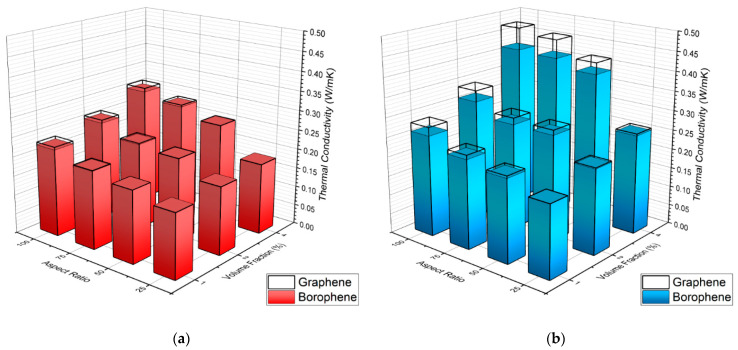
Effective thermal conductivity of PDMS nanocomposites as a function of nanofiller volume fraction and aspect ratio. The thickness of nanosheets was assumed to be (**a**) 1 nm and (**b**) 100 nm; 1 nm conductivities are superimposed.

We first studied the effects of volume fraction and aspect ratio on the effective thermal conductivity of nanocomposites, and the obtained results are shown in Figure 5. In these results, the thickness of nanosheets was assumed to be 1 nm (Figure 5a) and 100 nm (Figure 5b), taken as lower and upper bound values for experimentally exploitable nanoplates. All the predicted values for nanocomposites are clearly higher than the thermal conductivity of pure PDMS (0.15 Wm^−1^ K^−1^), demonstrating enhancement due to the presence of fillers with higher thermal conductivity. The volume fraction of the filler, as known from the literature, was confirmed as the typical parameter to tune in order to improve heat transfer in composite materials; 4% of filler about doubled the TC compared to 1%. As expected, by increasing the volume fraction and aspect ratio, the effective thermal conductivity of PDMS nanocomposites increased monotonically. By increasing the aspect ratio, the fillers allowed more direct heat transfer along the composite. As known from the literature [44], short particles featuring a low aspect ratio (1:25 and 1:50) confirmed to provide a smaller contribution than longer ones to improve thermal transport in composites. When particles with a low aspect ratio were employed, the relatively thick layer of polymer interposed within the particles did not allow particles to create a three-dimensional path able to transport heat efficiently. As a very interesting finding, it is clear that borophene nanosheets, despite having almost two orders of magnitude lower thermal conductivity than graphene, yield very close enhancement in the effective thermal conductivity in comparison with graphene, particularly for low content and small aspect ratio and thickness. For the nanocomposites with low concentration of fillers with small aspect ratios and thicknesses, borophene and graphene fillers showed similar enhancement ratios. Nonetheless, graphene-based nanocomposites always showed higher thermal conductivities than their borophene counterparts. This reveals that the slightly different thermal conductance between borophene and PDMS could never compensate for their lower thermal conductivity compared to graphene. The results shown in Figure 5 clearly highlight that the difference due to the type of nanofillers on the enhancement of thermal conductivity became more pronounced with higher volume fractions, thickness and aspect ratio for the nanofillers. When comparing the effects of thickness on the effective thermal conductivity, our results showed substantial effects. By increasing the thickness for a given volume fraction of conductive particles, the effect of interfacial resistance decreases, resulting in a higher thermal conductivity. This finding suggests that, for polymer-based composites, significant improvement in thermal conductivity can be reached by improving the bonding between the fillers and polymer, or in other words, enhancing the thermal conductance at the interface. It is noticeable that borophene nanofillers with 100 nm thickness could yield distinctly higher enhancement in the effective thermal conductivity of nanocomposites than those made of graphene nanosheets with a thickness of 1 nm. Our results reveal that nanofillers with higher thicknesses and aspect ratio can result in higher thermal conductivities. It is also clear that nanofillers with higher thermal conductivities become more effective when their aspect ratio and thickness are larger.

## 4. Concluding Remarks

In this study, a combined atomistic continuum multi-scale modeling approach was developed to explore the effective lattice thermal conductivity of polymer nanocomposites made of PDMS and graphene or borophene nanofillers. This approach includes an initial step where classical molecular dynamics simulations were employed to investigate the interfacial thermal conductance between graphene–polymer and borophene–polymer interfaces. In a following step, the estimated interfacial thermal conductances were used within the finite element method to evaluate the effective thermal conductivity of polymer nanocomposites in the continuum. In particular, we examined the effects of nanofiller type—borophene or graphene—and their volume content, geometrical aspect ratio and thickness. Based on the Molecular Dynamics calculations, the interfacial thermal conductance between the PDMS polymer and graphene or borophene were predicted to be 30 MWm^−2^ K^−1^ and 33 MWm^−2^ K^−1^, respectively. Acquired results confirm that the interfacial thermal conductance between nanosheets and polymer increases from the single-layer to the multilayered nanofillers and finally converges, in accordance with the adopted technique. These estimated converged values were then employed to define contact thermal conductance in the finite element modeling of nanocomposites’ representative volume elements. Taking into account the high electrical conductivity of borophene, our results therefore suggest borophene nanosheets as promising candidates for the improvement of polymers’ thermal and electrical conductivity. As a very interesting finding, we showed that borophene nanosheets, despite having almost two orders of magnitude lower thermal conductivity than graphene, yield very close enhancement in the effective thermal conductivity in comparison with graphene, particularly for low content and small aspect ratio and thickness. This finding confirms that, for polymer-based composites, thermal resistance between the fillers and matrix can dominate the heat transport. Thus, significant improvement in the thermal conductivity can be reached by improving the bonding between the fillers and polymer, or in other words, by enhancing the thermal conductance at the interface, for example, by chemical functionalization. Additionally, the possibility of manufacturing an effective percolation network through the exploitation of flake-to-flake junctions represents the most efficient and challenging route to improve the thermal conductivity of nanocomposites. The future development of theoretical models capable of managing flake-to-flake thermal conductance and chemical functionalization will reduce the gap between the experimental and the computational approaches, driving a more accurate generation of predictive studies.

## Data Availability

The data presented in this study are available on request from the corresponding authors.

## Abbreviations

PDMS	polydimethylsiloxane
MD	Molecular Dynamics
NVT	canonical ensemble (constant number of particles, volume and temperature)
NVE	microcanonical ensemble (constant number of particles, volume and energy)
FEM	finite element method
RVE	representative volume element, a (cube-shaped) portion of the composite
DC3D4	4-node linear heat transfer tetrahedron
